# The ventricular-subventricular zone: a source of oligodendrocytes in the adult brain

**DOI:** 10.3389/fncel.2014.00137

**Published:** 2014-05-16

**Authors:** Oscar Gonzalez-Perez

**Affiliations:** Laboratory of Neuroscience, School of Psychology, DES Ciencias de la Salud, University of ColimaColima, Mexico

**Keywords:** neural stem cells, ventricular-subventricular zone, oligodendrocyte, myelin, white matter, oligodendrocyte precursor cells, remyelination, demyelinating disease

Demyelinating diseases are characterized by an extensive loss of oligodendrocytes and myelin sheaths from axolemma, which commonly result in disability in young adults. To date, there is no effective treatment against these neurological disorders. In the adult brain, there are neural stem cells (NSCs) that reside within a niche denominated ventricular-subventricular zone (V-SVZ) in the lateral wall of the cerebral ventricles. These NSCs give rise to neurons and oligodendrocytes that help preserve cellular homeostasis (Kriegstein and Alvarez-Buylla, 2009). Growing evidence indicates that V-SVZ progenitor cells may represent an endogenous source of oligodendrocytes that can be useful to treat demyelinating diseases.

The e-Book “*The ventricular-subventricular zone as a source of oligodendrocytes in the adult brain*” has collected the most recent evidence regarding the mechanisms that modulate the proliferation, migration, quiescence, cell-fate choices and survival of oligodendrocyte precursors generated in the V-SVZ. This e-Book begins with an excellent study performed by the members of the Nada Zecevic's laboratory. They analyzed the effects of Sonic hedgehog (Shh) signaling on proliferation and specification of human cortical Olig2+ progenitors *in vitro*. They demonstrated that Shh increased the number of oligodendrocyte progenitors (OPCs). However, inhibition of endogenous Shh did not reduce the density of Olig2+ cells, which suggest an additional Shh-independent mechanism for oligodendrocyte generation (Ortega et al., 2013).

In the adult V-SVZ, the activation of NMDA receptors (NMDAR) increases the oligodendrocyte differentiation. Using the reporter gene luciferase, Carlos Matute et al. (Chapter 2) found that the activation of NMDR stimulates PKC, which precedes the activation of NADPH oxidase (NOX) (Cavaliere et al., 2013). Hence, the authors propose that NOX2 is involved in the transduction of the signal from NMDAR through PKC activation. This work also suggests that signaling through the cascade NMDAR–PKC-NOX2 generates ROS that, in turn, activate the PI3/mTOR pathway and drives oligodendrogenesis.

Tyrosine kinase (Trk) receptors play a key role in regulating the function of V-SVZ NSCs. Particularly, V-SVZ neural progenitor cells express the epidermal growth factor receptor (EGFR). EGF strongly stimulates proliferation, survival, migration and differentiation into the oligodendrocyte lineage (Gonzalez-Perez and Alvarez-Buylla, 2011). In the chapter 3, Galvez-Contreras and colleagues compiled the current data regarding the role of EGFR and ErbB family signaling on V-SVZ NSCs and explained the downstream cascades involved in oligodendrogenesis (Galvez-Contreras et al., 2013). The authors also proposed a hypothetical model to support the EGF-induced oligodendrogenesis, which involves homodimerization between ErbB1 and ErbB3, which probably activate the PI3K or the STAT pathways. Inconsequence AKT is activated and the expression of Olig-2 is induced, which determines oligodendroglial specification.

Endocannabinoids have been involved in oligodendrogenesis. As reported by the Dr. Malva's group (chapter 4), hemopressin, a modulator of cannabinoid receptor-1, can increase the oligodendroglial differentiation in V-SVZ progenitor cells (Xapelli et al., 2014). Their results suggest that hemopressin may be of potential interest to treat demyelinating diseases.

Erythropoietin (EPO) promotes the V-SVZ-derived neurogenesis and oligodendrogenesis. Sawamoto and coworkers recently demonstrated that the EPO derivative asialo-EPO promotes the differentiation of V-SVZ-derived OPCs into myelin-forming mature oligodendrocytes in the injured white matter of neonatal mice without causing erythropoiesis (Kaneko et al., 2013). These findings and the therapeutic proposal are discussed in the chapter 5 of this book.

In the chapter 6, the group of Prof. Dietzel found that the addition of equiosmolar supplements of mannitol or NaCl associated with microglia cells can influence the proliferation of OPCs (Kleinsimlinghaus et al., 2013). Interestingly, a maximal yield of OPCs is obtained by combining a cell culture medium with osmolarity >280 mOsm and low density of microglia cells.

Aging progressively decline in the proliferative capacity of V-SVZ progenitor cells that subsequently affects the incorporation of new cells in the olfactory bulb. In the chapter 7, Quiñones-Hinojosa's group presents the effects of aging across the V-SVZ-OB system. They surprisingly found that neurogenesis decline with aging, but oligodendrogenesis in the rostral migratory stream is not compromised (Capilla-Gonzalez et al., 2013).

In the chapter 8, Ken Arai and colleagues (Maki et al., 2013) summarized the recent studies on extrinsic (extracellular matrix, cerebrospinal fluid, vasculature) and intrinsic (transcription factors or epigenetic modifiers) factors, which mediate oligodendrocyte generation from the V-SVZ progenitor cells. These factors appear to decrease by aging and compromise the efficiency of remyelination into the brain. In the chapter 9, significant questions and hypotheses regarding this topic are analyzed by Agathou et al. (2013). In the last chapter, Zhang et al. compiled evidence that indicates that a pathological condition as stroke can increase oligodendrogenesis in the white matter and V-SVZ during brain repair (Zhang et al., 2013), which support the notion that many inflammatory cytokines regulate the V-SVZ cells (Gonzalez-Perez et al., 2012).

In summary, growing evidence indicates that V-SVZ NSCs are good candidates to establish stem cell-based therapies in demyelinating disorders. Nevertheless, chemical mediators and signaling pathways involved in all these processes are to be completely elucidated. Understanding the mechanisms that regulate the V-SVZ progenitor cells may help develop therapeutic approaches to treat demyelinating diseases.

## Conflict of interest statement

The author declares that the research was conducted in the absence of any commercial or financial relationships that could be construed as a potential conflict of interest.

## References

[B1] Agathou S. Karadottir R. T. Kazanis I. (2013). Niche derived oligodendrocyte progenitors: a source of rejuvenation or complementation for local oligodendrogenesis? Front. Cell. Neurosci. 7:188 10.3389/fncel.2013.00188 24155691 PMC3804763

[B2] Capilla-Gonzalez V. Cebrian-Silla A. Guerrero-Cazares H. Garcia-Verdugo J. M. Quinones-Hinojosa A. (2013). The generation of oligodendroglial cells is preserved in the rostral migratory stream during aging. Front. Cell. Neurosci. 7:147 10.3389/fncel.2013.00147 24062640 PMC3775451

[B3] Cavaliere F. Benito-Munoz M. Panicker M. Matute C. (2013). NMDA modulates oligodendrocyte differentiation of subventricular zone cells through PKC activation. Front. Cell. Neurosci. 7:261 10.3389/fncel.2013.00261 24391542 PMC3866621

[B4] Galvez-Contreras A. Y. Quinones-Hinojosa A. Gonzalez-Perez O. (2013). The role of EGFR and ErbB family related proteins in the oligodendrocyte specification in germinal niches of the adult mammalian brain. Front. Cell. Neurosci. 7:258 10.3389/fncel.2013.00258 24381541 PMC3865447

[B5] Gonzalez-Perez O. Alvarez-Buylla A. (2011). Oligodendrogenesis in the subventricular zone and the role of epidermal growth factor. Brain Res. Rev. 67, 147–156 10.1016/j.brainresrev.2011.01.001 21236296 PMC3109119

[B6] Gonzalez-Perez O. Gutierrez-Fernandez F. Lopez-Virgen V. Collas-Aguilar J. Quinones-Hinojosa A. Garcia-Verdugo J. M. (2012). Immunological regulation of neurogenic niches in the adult brain. Neuroscience 226, 270–281 10.1016/j.neuroscience.2012.08.053 22986164 PMC3490038

[B7] Kaneko N. Kako E. Sawamoto K. (2013). Enhancement of ventricular-subventricular zone-derived neurogenesis and oligodendrogenesis by erythropoietin and its derivatives. Front. Cell. Neurosci. 7:235 10.3389/fncel.2013.00235 24348331 PMC3842008

[B8] Kleinsimlinghaus K. Marx R. Serdar M. Bendix I. Dietzel I. D. (2013). Strategies for repair of white matter: influence of osmolarity and microglia on proliferation and apoptosis of oligodendrocyte precursor cells in different basal culture media. Front. Cell. Neurosci. 7:277 10.3389/fncel.2013.00277 24421756 PMC3872727

[B9] Kriegstein A. Alvarez-Buylla A. (2009). The glial nature of embryonic and adult neural stem cells. Annu. Rev. Neurosci. 32, 149–184 10.1146/annurev.neuro.051508.135600 19555289 PMC3086722

[B10] Maki T. Liang A. C. Miyamoto N. Lo E. H. Arai K. (2013). Mechanisms of oligodendrocyte regeneration from ventricular-subventricular zone-derived progenitor cells in white matter diseases. Front. Cell. Neurosci. 7:275 10.3389/fncel.2013.00275 24421755 PMC3872787

[B11] Ortega J. A. Radonjic N. V. Zecevic N. (2013). Sonic hedgehog promotes generation and maintenance of human forebrain Olig2 progenitors. Front. Cell. Neurosci. 7:254 10.3389/fncel.2013.00254 24379757 PMC3861791

[B12] Xapelli S. Agasse F. Grade S. Bernardino L. Ribeiro F. F. Schitine C. S. (2014). Modulation of subventricular zone oligodendrogenesis: a role for hemopressin? Front. Cell. Neurosci. 8:59 10.3389/fncel.2014.00059 24578683 PMC3936357

[B13] Zhang R. Chopp M. Zhang Z. G. (2013). Oligodendrogenesis after cerebral ischemia. Front. Cell. Neurosci. 7:201 10.3389/fncel.2013.00201 24194700 PMC3810592

